# Stem Cells in Skin Regeneration, Wound Healing, and Their Clinical Applications

**DOI:** 10.3390/ijms161025476

**Published:** 2015-10-23

**Authors:** Nkemcho Ojeh, Irena Pastar, Marjana Tomic-Canic, Olivera Stojadinovic

**Affiliations:** 1 Faculty of Medical Sciences, the University of the West Indies, Cave Hill Campus, P.O. Box 64, Bridgetown BB 11000, St. Michael, Barbados; E-Mail: nkemcho.ojeh@cavehill.uwi.edu; 2 Wound Healing and Regenerative Medicine Research Program, Department of Dermatology and Cutaneous Surgery, University of Miami Miller Medical School, 1600 NW 10th Avenue, RMSB, Room 2023-A, Miami, FL 33136, USA; E-Mails: ipastar@med.miami.edu (I.P.); mtcanic@med.miami.edu (M.T.-C.)

**Keywords:** skin, epidermis, stem cells, wound healing, chronic wounds

## Abstract

The skin is the largest organ of the body and has an array of functions. Skin compartments, epidermis, and hair follicles house stem cells that are indispensable for skin homeostasis and regeneration. These stem cells also contribute to wound repair, resulting in restoration of tissue integrity and function of damaged tissue. Unsuccessful wound healing processes often lead to non-healing wounds. Chronic wounds are caused by depletion of stem cells and a variety of other cellular and molecular mechanisms, many of which are still poorly understood. Current chronic wound therapies are limited, so the search to develop better therapeutic strategies is ongoing. Adult stem cells are gaining recognition as potential candidates for numerous skin pathologies. In this review, we will discuss epidermal and other stem cells present in the skin, and highlight some of the therapeutic applications of epidermal stem cells and other adult stem cells as tools for cell/scaffold-based therapies for non-healing wounds and other skin disorders. We will also discuss emerging concepts and offer some perspectives on how skin tissue-engineered products can be optimized to provide efficacious therapy in cutaneous repair and regeneration.

## 1. Introduction

The skin, the largest organ in the body, possesses an array of functions—acting as a barrier for protection and prevention of dehydration, as a sensory and thermoregulatory organ, and as an active site of vitamin D synthesis and immune surveillance [1]. The skin is comprised of the epidermis and the dermis. Morphologically, the epidermis is arranged into distinct layers that reflect the sequential differentiation of keratinocytes as they migrate from the basal layer on the onset of terminal differentiation, having lost the ability to proliferate, to the outermost cornified layers, where they are sloughed off [2,3]. The epidermis and hair follicle are maintained and regenerated through the existence of stem cells [4]. Epidermal stem cells are relatively quiescent and undifferentiated with a capacity to maintain homeostasis, self-renew tissue, and contribute to wound repair.

Skin wound healing is a highly organized and coordinated series of processes that results in the restoration of tissue integrity and functions. An interruption in the normal wound healing process can lead to the development of non-healing chronic wounds. A number of factors can cause a delay in wound healing including venous or arterial insufficiency, diabetes, renal disease, trauma, advanced age, and local pressure effects. Local factors like tissue hypoxia, ischemia, foreign bodies, maceration of tissue, exudates, infection, disruption of the regulation of the inflammatory process, and systemic factors including compromised nutritional or immune status can all impair healing (reviewed in [5]). It is important to note that the increased prevalence of non-communicable diseases such as diabetes, obesity, and vascular disease is a contributing factor to the rise of chronic wounds. Such wounds as diabetic, venous, and pressure ulcers are creating a major global issue with significant management costs. In the United States alone, more than 6 million people are afflicted with chronic wounds, which is placing a major burden on the health care system, with an estimated annual cost of $25 billion [6,7]. Fifteen percent of diabetic patients suffer from diabetic foot ulcers (DFUs), many of these leading to lower-leg amputations [5,8]. Several therapies have been developed for chronic wounds, with varying degrees of success [5,9]. Accumulating experimental evidence suggests that the use of stem cells as a potential wound therapy is gaining widespread recognition.

This review will focus primarily on epidermal and other skin stem cells, their therapeutic applications as tools for cell/scaffold-based therapies for non-healing wounds, and, to a lesser extent, other skin disorders. In the following sections, we will define epidermal stem cells and summarize some of the biomarkers used for their identification, as well as some associated genes and signaling pathways that regulate their fate and activity based on reported research. We will focus on the relevance of epidermal stem cells and other adult stem cells in the context of wound healing and skin disorders, and discuss their potential application in cell/scaffold-based wound therapies as well as their limitations.

## 2. Epidermal Stem Cells

Adult stem cells reside in specific microenvironments called niches that are important for modulating stem cell fate and activity [10]. Three distinct epidermal stem cell niches have been identified in the skin. These are the basal layer of the epidermis, the “bulge region” of the hair follicle, a morphologically distinct region in mice, but not in humans, and the base of the sebaceous glands [11,12]. Under steady-state conditions, each discrete niche maintains its respective tissue compartment in a unipotent fashion; this has been confirmed by fate-mapping and live-imaging studies [13,14]. In skin, different theories exist to explain the maintenance of epidermal homeostasis. The classical hierarchical model proposes that slow cycling stem cells divide in the basal layer and give rise to transit amplifying cell (TA) daughters that amplify the number of cells for the replenishment of the tissue. They undergo a finite number of cell divisions before becoming terminally differentiated as they transit upwards through the suprabasal layers. According to this model, stem cells and their progeny are organized in an epidermal proliferative unit (EPU) in the mouse epidermis, where the slow-cycling stem cells are found at the center of the EPU, and the more proliferative, TA cells are found at the periphery of the EPU [15,16]. Recently, however, this model has been challenged by a new stochastic model of homeostasis involving only one type of progenitor cell that may undergo an unlimited round of divisions to give rise to two terminally differentiating cells, two undifferentiated basal cells, or one of each type [17,18]. Findings obtained from recent studies support both models [17,19,20]; this may be due to epidermal variation at different anatomical locations [18].

In hair follicles two main subpopulations of stem cells are believed to exist, a subset located within the hair germ just below the bulge that gives rise to the hair shaft and inner root sheath (IRS) and the quiescent group, which resides in the bulge region that gives rise to the basal outer root sheath (ORS) keratinocytes. Although the bulge region is the most well-defined stem cell niche in the skin [4] owing to its slow cycling, quiescent nature [21], clonogenic capability [22], and the expression of a subset of markers [23], recent studies have reported multiple partly overlapping stem cell populations outside of this anatomical region that have differing abilities to contribute to the interfollicular epidermis (IFE), hair follicle, and sebaceous gland [24]. In mice, these cells express a number of characterized markers. For instance, cells expressing keratin 15 (K15), a leucine-rich repeat containing G protein-coupled receptor 5 (Lgr5), CD34, and SRY box 9 (Sox9), are found in the bulge region [25,26,27,28]. B-lymphocyte-induced maturation protein 1 (Blimp1)-positive cells are thought to mark sebaceous gland stem cells [29]; Placenta-expressed transcript 1 (Plet1)/MTS24 and Lrig1-expressing stem cells reside in the upper isthmus/junctional zone region [29,30,31] and Lgr6- and Glioma-associated oncogene homolog 1 (Gli1)-positive cells are located in the lower isthmus [32,33]. Furthermore, other genes that regulate ESCs and their fate in the HF bulge include GATA binding protein 3 (GATA3), bone morphogenetic protein receptor1a (BMPR1a), and the inhibitors of DNA-binding protein 2 and 4 (ID2, ID4), Wnt and β-catenin [34,35,36]. Human hair follicle bulge stem cells express K15, pleckstrin homology-like domain, family A member 1 (PHLDA1) [37], cluster of differentiation 200 (CD200) [38], and K19 [39], although the latter is also expressed in the suprabulbar ORS in human anagen follicle [40] and in the basal layer of the IFE [39,41]. Recently Lgr6-positive and Bmi1-positive stem cells were found to be responsible for maintaining the acral epithelium by maintaining sweat glands, ducts, and the inter-adnexal epidermis, thus facilitating the regeneration of these structures following injury [42] (Table 1).

**Table 1:** Some examples of epidermal and hair follicle stem cells and their locations and markers.

Stem Cells	Location (Niche)	Markers
Interfollicular epidermal stem cells	Epidermal basal layer	p63, β1^high^/melanoma chondroitin sulfate proteoglycan + (MCSP+), α6^high^/CD71^dim^
Hair follicle stem cells	Bulge region	K15, CD34, Lgr5, Sox9, Lhx2, NFATC1, NFIB, K15, PHLDA1, CD200, K19, bromodeoxyuridine dye retention
Hair follicle stem cells	Isthmus	Lrig1, MST24, Lgr6, Gli1
Hair follicle stem cells	Hair germ at base of hair follicle	K15, Lgr5, Gli1
Sebaceous gland stem cells	Sebaceous glands, infundibulum	Blimp1
Melanocyte stem cells	Hair follicle bulge region and hair germ	Dct, Sox, Pax3
Neural progenitor cells	Bulge region	Nestin

### 2.1. Epidermal Cell Clonal Conversion

Over the past decade, significant progress has been made in identifying specific markers for stem cell/progenitor cell isolation and enrichment. This has been made possible through use of mouse models, techniques such as transplantation, lineage-tracing, label retaining, and fate-mapping studies, and functional assays like *in vitro* colony formation. Through such cell culture techniques, it has been shown that epidermal keratinocytes are a heterogeneous population with regards to their clonogenicity [43,44]. Using morphological criteria, three types of colonies, holoclones, paraclones, and meroclones are produced from single keratinocytes based on their proliferative potential. Holoclones are large and circular and contain small, regularly shaped cells with the greatest proliferative potential. These colonies, thought to be formed of stem cells, express high levels of β1 integrin, K14, and p63 [45,46,47], have self-renewing abilities, and give rise to both meroclones and paraclones [44]. Meroclones, believed to be TA cells, contain a mixture of cells with varying growth potential, giving rise to both paraclones and meroclones when re-seeded [43]. Levels of p63 expression by meroclones were shown to fall dramatically as they evacuate from the stem cell niche [46]. Paraclones form small irregular shaped colonies and are believed to be post-mitotic committed cells. These cells only possess a short replicative life span and express high levels of the terminal differentiation marker, involucrin [43]. The transition from holoclone to meroclone to paraclone is known as clonal conversion and is irreversible under normal circumstances.

### 2.2. Epidermal Stem Cells Engage in Tissue Repair Following Injury

In response to injury, stem cells from the hair follicle and IFE contribute towards re-epithelialization of wounds [48,49,50]. In full-thickness wounds, cells from hair follicles and IFE have been shown to migrate to the wound site [49,51,52,53]. Fate-mapping experiments demonstrated that K15-positive hair follicle bulge stem cells transiently contribute to wound re-epithelialization in full-thickness wounds in mice soon after injury but were lost from the epidermis several weeks later, suggesting that stem cells from the hair follicle are not mandatory for the long-term upkeep of the IFE but contribute during wound healing [49]. In support of this, Langton *et al.* [54] demonstrated a delay in the early stages of re-epithelialization, eventually leading to complete epidermal closure in linear incisional wounds of the tail skin of mutant mice lacking hair follicles, presumably by IFE stem cells indicating their capability for tissue regeneration. Gli1+, Lrig1+, Lrg5+, and MT24+ cells have all been shown to contribute to the homeostasis of the pilosebaceous unit and, in response to skin injury, become activated and contribute towards IFE repair [30,32,33,53,55,56], demonstrating the plasticity of epidermal stem cells. Clinical evidence also suggests that hair follicle progenitor cells can contribute to the re-epithelialization of wounds [57]. Jimenez *et al.* [57] evaluated the feasibility and potential healing capacity of autologous scalp follicular grafts transplanted into the wound bed of chronic leg ulcers in 10 patients in a pilot study and reported a 27.1% ulcer area reduction in the experimental square compared to 6.5% in the control square by 18 weeks. Epithelialization, neovascularization, and dermal reorganization were also enhanced within these wounds, highlighting the feasibility of hair follicle grafting as a promising therapeutic alternative for non-healing chronic wounds. In another study, the implantation of hair follicle micrografts into a collagen-glycosaminoglycan neodermis on a full-thickness scalp burn gave rise to a normal multilayered, differentiated epidermis derived from ORS cells [58]. At the same time, it has been shown that these hair follicle progenitor cells are largely replaced by epidermal progeny following repair [51]. Indeed, in studies where laser ablation of bulge stem cells was performed, cells from the upper hair follicle region and IFE were capable of replacing the bulge stem cells [59]. These findings thus indicate that both IFE and hair follicle stem cells participate in wound healing but the latter are not necessary for the long-term maintenance of the IFE.

### 2.3. MicroRNAs as Regulators of Epidermal Stem Cell Maintenance and Wound Healing

MicroRNAs (miRNAs) are small, noncoding RNAs that regulate gene expression post transcriptionally by repressing messenger RNA (mRNA) translation or inducing their degradation [60]. One miRNA is capable of targeting hundreds of genes while one gene can be regulated by multiple miRNAs [61]. As central regulators of gene expression, miRNAs play key roles in many biological processes including cell survival, homeostasis, and differentiation, while their aberrant expression can lead to development of disease [62,63]. Their role in epidermal development and adult skin stem cell maintenance has been well documented [64,65].

Several miRNAs were identified that were expressed differentially or exclusively in the mice epidermis compared with other skin lineages [66]. The miR-200 family (a, b, and c), miR-141, miR-429, and the miR-19/miR-20 family (miR-19b, miR-20, miR-17-5p, and miR-93) were expressed in epidermal lineage, whereas the miR-199 family was present only in hair follicles [65,66]. In a recent study, Hildebrand *et al.* [67] compared the expression profiles of calcium-induced differentiated keratinocytes with those of miRNA of epidermal stem cells, TA keratinocytes and terminally differentiated cells isolated from human skin. They reported eight upregulated miRNAs in differentiated keratinocytes (miR-23b, miR-95, miR-210, miR-224, miR-26a, miR-200a, miR-27b, and miR-328), and one downregulated miRNA (miR-376a) both *in vivo* and *in vitro*, suggesting that they are involved in epidermal differentiation. miR-203 plays an important role in skin morphogenesis and keratinocyte differentiation, and represses “stemness” by inhibiting the suprabasal expression of p63 [68,69], a transcription factor essential for the initiation of epithelial stratification and maintenance of proliferation of basal keratinocytes [70]. During acute wound healing, miR-203 expression was downregulated in the leading edge of the epithelial migrating tongue along with upregulated expression of p63, RAN (member of the G-protein superfamily), and RAPH1 (lamellipoidin), indicating its contribution to wound epithelialization [71]. Conversely, chronic venous ulcers showed increased expression of miR-203 [72]. An antiproliferative effect of miR-483-3p in human keratinocytes has also been reported in scratch wound assays of human keratinocytes and wounded skin in mice [73]. Moreover, studies have also shown the importance of miR-125b in epithelial stem cell regulation. Expression of miR-125b is increased in the “stem” state but downregulated in early skin stem cell progeny [74]. Additionally, dysregulation of miR-125b contributes to hyperproliferation in the psoriatic epidermis. Overexpression of miR-125b in primary human keratinocytes suppresses proliferation, while its knockdown induces proliferation and delays differentiation via fibroblast growth factor receptor 2 (FGFR2) regulation [75].

MiRNAs can also be secreted by a variety of cell types and transported via exosomes into the circulation [76], where they modulate cellular activity of target cells and are involved in cell-to-cell communication [77,78,79]. Recently, exosomes derived from bone marrow-derived mesenchymal stem cells (BMSCs) were shown to be enriched in distinctive miRNAs [80] capable of enhancing proliferation and migration of normal and diabetic wound fibroblasts [78]. Exosomes released by keratinocytes were shown to modulate melanin synthesis by melanocytes [77]. Furthermore, Mistry *et al.* reported that several subunits of exosomes were enriched in epidermal progenitor cells that were important for retaining their proliferative potential and preventing their premature differentiation [81]. Findings from these studies provide further evidence for the role of miRNAs, partly mediated by exosomes in maintenance of epidermal stem cells.

## 3. Evidence for Epidermal Stem Cell Survival *in Vitro* and Their Clinical Application

Several studies have confirmed the survival of epidermal stem cells in *in vitro* culture [58,82,83]. Dunnwald *et al.* [82] used specifically defined gating methods to distinguish three populations of mouse epidermal cells: stem cells, TA cells, and non-proliferative basal cells. When used in conjunction with a collagen type I gel seeded with dermal fibroblasts, only the stem cell population was able to form and maintain a normal epidermis for up to six months. Further evidence comes from the use of cultured keratinocyte sheets, also known as cultured epithelial autografts (CEA), derived from skin. Under optimal conditions, keratinocytes derived from a 3-cm^2^ skin biopsy can be expanded to generate large, multilayered CEA within three to four weeks in culture, enough to cover the whole body [84,85]. These have been used as epidermal substitutes and successfully engrafted in burn victims, where they have been shown to produce a normal epidermis that persists for several years [83].

Epidermal substitutes, some of which are commercially available, come in various forms such as confluent or preconfluent autologous or allogeneic keratinocytes on delivery systems, or cells used in conjunction with aerosol spray methods to facilitate delivery to the wound (see examples in Table 2). Some examples of these include EpiCel and EpiDex [86]. In a retrospective study, EpiDex, comprised of ORS keratinocytes derived from scalp hair follicles, was used to treat chronic wounds and led to healing of three-quarters of recalcitrant chronic leg ulcers [87]. Cryoskin is an example of allogeneic keratinocyte sheets [88]. To limit any delays in *in vitro* expansion and maintain a proliferative phenotype, cells have also been grown to preconfluency on delivery systems that facilitate transplantation such as Laserskin^®^ and Myskin [89,90]. The use of fibrin glue in conjunction with the aerosol method for keratinocyte delivery of cell suspension to wounds in the form of BioSeed-S and CellSpray has also been reported [84,91]. The successful clinical application of these epidermal substitutes for the treatment of venous and diabetic ulcers, and burn wounds has been described in a variety of studies [92,93,94].

**Table 2:** Some examples of skin scaffolds: epidermal substitutes (**A**); dermal substitutes (**B**); composite substitutes (**C**) [95].

**A**			
**Product Name**	**Company**	**Description**	**Uses**
*Epidermal Substitutes*			
Epicel	Genzyme Corp., Cambridge, MA, USA	Confluent cultured autologous keratinocyte sheet delivered on petroleum gauze backing	Burn wounds, acute wounds; chronic wounds
Cryoskin	Altrika Ltd., Sheffield, UK	Confluent cultured allogenic keratinocyte sheet on silicone backing	Burn wounds, chronic wounds; donor site wounds
CellSpray	Avita Medical, Northridge, CA, USA	Subconfluent suspension of proliferative keratinocytes applied to wounds via spraying	Partial thickness wounds; donor site wounds
EpiDex	Modex Therapeutics, Lausanne, Switzerland	Confluent cultured autologous keratinocyte sheet from ORS cells from hair follicles on silicone membrane	Full-thickness wounds; burn wounds; chronic wounds
MySkin	Altrika Ltd., Sheffield, UK	Subconfluent cultured autologous keratinocytes grown on silicone support treated with plasma polymer film	Partial-thickness wounds; burn wounds; chronic wounds; donor-site wounds
Celaderm	Celadon Science LLC, Brookline, MA, USA	Living foreskin-derived allogenic keratinocytes	Partial and full-thickness wounds; burn wounds, chronic wounds
BioSeed-S	BioTissueTechnologies, Freiburg, Germany	Autologous keratinocytes in fibrin glue	Burn wounds; chronic wounds
Biobrane	Smith & Nephew, Hull, UK	Bilaminar membrane with silicone layer bonded to nylon coated with peptides derived from porcine collagen type I	Partial-thickness and full-thickness wounds; burn wounds; donor site wounds
Suprathel	Stapleline GmbH, Bochum, Germany	Acellular synthetic co-polymer based on DL-lactide and contains triethylenecarbonate and ε-caprolactone	Burn wounds; donor site wounds
Laserskin	Fidia Advanced Biopolymers, Abano Terme, Italy	100% esterified hyaluronic acid membrane with laser drilled micropores seeded with autologous keratinocytes	Partial-thickness wounds; burn wounds, chronic wounds; vitiligo treatment
**B**			
**Product Name**	**Company**	**Description**	**Uses**
*Dermal Substitutes*			
AlloDerm	LifeCell Corporation, The Woodlands, TX, USA	Human allogenic acellular dermis (cadaveric)	Full-thickness and burn wounds; chronic wounds; reconstruction
Hyalomatrix	Fidia Advanced Biopolymers, Abano Terme, Italy	Hyaluronic acid matrix with variable esterification attached to silicone membrane as temporary epidermis	Burns, acute and chronic wounds
Hyalograft-3D	Fidia Advanced Biopolymers, Abano Terme, Italy	Esterified hyaluronic acid matrix with autologous fibroblasts attached to silicone membrane as temporary epidermis	Partial- and full-thickness wounds; burns, acute; chronic wounds
Dermagraft	Organogenesis Inc., Canton, MA, USA	Bioabsorbable polyglactin mesh with living cultured allogenic neonatal foreskin-derived fibroblasts	Full-thickness wounds; burn wounds; chronic wounds; epidermolysis bullosa
Integra	Integra LifeSciences Corporation, Plainsboro, NJ, USA	Bovine collagen type I and shark chondroitin-6-sulphate attached to silicone membrane as temporary epidermis	Full-thickness wounds; burns wounds; acute wounds; chronic wounds
Matriderm	Medskin Solutions, Billerbeck, Germany	Acellular scaffold composed of bovine collagens types I, II, V, and elastin	Full-thickness wounds; burn wounds
Strattice	LifeCell Corporation, Bridgewater, NJ, USA	Allogenic porcine acellular dermis	Reconstruction
Trancyte	Advanced BioHealing, Inc., Westport, CT, USA	Nylon mesh coated with porcine collagen with non-viable cultured neonatal foreskin-derived fibroblasts attached to silicone membrane as temporary epidermis	Partial-thickness and full-thickness wounds; burn wounds
Ez-Derm	Brennen Medical, Inc., St. Paul, MN, USA	Perforated or non-perforated cross-linked porcine collagen	Partial-thickness wounds; burn wounds; chronic wounds
EpiFix	MiMedx Group Inc., Marietta, GA, USA	Human amniotic membrane	Full-thickness wounds; acute wounds; chronic wounds
Oasis	Smith & Nephew, Hull, UK	Porcine small intestinal submucosa	Partial-thickness, full-thickness; acute and chronic wounds
**C**			
**Product Name**	**Company**	**Description**	**Uses**
*Composite Skin Equivalents*			
Apligraf	Organogenesis Inc., Canton, MA, USA	Bilayered matrix composed of bovine collagen type I with living cultured allogenic neonatal foreskin-derived fibroblasts and keratinocytes	Full-thickness wounds, burn wounds; acute wounds; chronic wounds; donor site wounds
OrCel	Forticell Bioscience, Inc., New York, NY, USA	Bilayered matrix composed of Bovine collagen type I with living cultured allogenic epidermal keratinocytes and dermal fibroblasts	Full-thickness wounds; burn wounds; chronic wounds; donor site wounds; epidermolysis bullosa
TissueTech	Fidia Advanced Biopolymers, Abano Terme, Italy	Hyalograft 3D and Laserskin combination	Chronic wounds
Theraskin	Soluble Systems, Newport News, VA, USA	Human cadaveric allograft skin containing donor fibroblasts and keratinocytes	Chronic wounds
StrataGraft	Stratatech Corporation, Madison, WI, USA	Dermal equivalent containing human dermal fibroblasts and stratified epidermis derived from genetically-stable, non-tumorigenic human keratinocyte progenitors, NIKS cells	Burn wounds; chronic wounds

As epidermal stem cells have the potential to regenerate skin, they offer a convenient vehicle for genetic manipulation and offer a great novel treatment option. A phase I clinical trial involving the application of autologous epidermal sheets comprised of corrected keratinocytes with wild-type collagen type VII, delivered by retroviral infection, in patients with recessive dystrophic epidermolysis bullosa, a genetic blistering disorder caused by mutations in the COL7A1 gene that leads to chronic wounds, is currently ongoing [96,97]. Robbins *et al.* [98] further demonstrated that the transfection of keratinocytes from patients suffering from junctional epidermolysis bullosa led to successful creation of phenotypically normal skin on severe combined immune deficient (SCID) mice. Mavilio *et al.* [99] also reported that the transduction of primary keratinocytes with laminin B3 cDNA from a patient suffering from junctional epidermolysis bullosa led to successful completion of epidermal regeneration on both legs on the patient throughout a one-year follow-up. Clearly, stem cell therapy is a developing therapy and has been shown to be beneficial for both acquired and inherited disorders, thus holding great promise in the treatment of devastating and difficult-to-treat skin diseases.

## 4. Other Hair Follicle Stem Cells

In humans, other subpopulations of stem cells reside within the hair follicle such as melanocyte stem cells, mesenchymal-like stem cells derived from the dermal sheath (DS) and dermal papilla (DP), and nestin-positive stem cells.

### 4.1. Dermal Papilla and Dermal Sheath Cells

The mesenchymal portions of the hair follicle are comprised of the DP, an almond-shaped structure surrounded by the follicle bulb epithelium containing mesoderm-derived fibroblasts, and the DS, which is contiguous with the DP. Both DP and DS have been shown to play pivotal roles in hair follicle formation, growth, and support [100,101,102,103]. Multipotent stem cells within the dermis have been reported to originate in the DP and DS [104] that can differentiate into smooth muscle cells, neurons, glial cells, and adipocytes [105]. Further, DS and DP cells have been shown to display adipogenic and osteogenic differentiation *in vitro* [106] and hematopoietic activity *in vivo* and *in vitro* [107]. It has been hypothesized that DP and DS cells may be a progenitor fibroblast population and participate in dermal repair during wound healing [108]. The transplantation of male-derived DS onto female skin wounds was reported to lead to the production of new follicles and fibers in a human model without any apparent sign of rejection [109].

### 4.2. Melanocyte Stem Cells

Melanocyte stem cells are located in the hair follicle bulge region and in the hair germ and express dopachrome tautomerase (Dct), Sox10, and paired box 3 (Pax3) [110,111,112,113]. These stem cells generate mature melanocytes present in the hair follicle bulb. During the anagen phase of the hair growth, mature melanocytes synthesize melanin from tyrosine via an enzymatic cascade controlled by tyrosinase-related protein 1 (TRP1), tyrosinase (TYR), and dopachrome tautomerase/tyrosinase-related protein 2 (Dct/TRP2). These genes are the targets of microphthalmia-associated transcription factor (MITF), a master regulator of pigmentation [112,114]. Melanin is responsible for hair pigmentation and is transferred by melanosomes to neighboring keratinocytes. Several studies have demonstrated that regulation of melanocyte stem cells occurs through cell–cell interaction in epidermal stem cells and via Wnt signaling [115], TGF-β [116], notch signaling [117], the transcription factor nuclear factor I/B (NFIB) [118], and the transmembrane protein Col17a1 [119]. Under normal steady state homeostasis, melanocytes are present in the IFE in human skin and solely in the hair follicles in adult mouse skin [120]. However, in response to cutaneous injury or ultraviolet B (UVB) exposure, melanocyte stem cells have been shown to migrate upwards to the IFE in a melanocortin 1 receptor (Mc1r)-dependent manner, where they differentiate into functioning epidermal melanocytes to protect the skin against injury [121].

### 4.3. Nestin-Positive Progenitor Cells

The protein marker for neural progenitor cells, nestin, is also expressed in the hair follicle [122]. Nestin-positive stem cells originate from the bulge region and migrate to the DP and surrounding tissue. These cells have a broad differentiated potential into various cell lineages such as neural, hepatic, pancreatic endocrine, cardiac muscle cell, and mesenchymal/mesodermal cell lineages [123,124,125,126,127]. Using green fluorescent protein (GFP) tracing techniques, labeled nestin-expressing hair follicle stem cells in mice were shown to have the capability to differentiate into multiple lineages such as keratinocytes, melanocytes, neurons, glial cells, and smooth muscle cells [123,128]. Furthermore, implanting these stem cells into the gap region of severed sciatic nerve stimulated nerve regeneration and restored nerve function, thus pointing to sophisticated interaction between skin, its appendage, and neuronal stem cells [128].

## 5. Induced Pluripotent Stem Cells

Differentiated, adult somatic cells (e.g., human skin keratinocytes, mouse and human fibroblasts, lymphocytes, liver cells) can be reprogrammed to generate induced pluripotent stem cells (iPSCs) with similar characteristics to embryonic stem cells [129,130,131,132]. This can be achieved by exogenous addition of four transcription factors (Oct-3/4, Sox2, c-Myc, and Klf4) using retroviral transduction. IPSCs have been shown to generate a wide range of differentiated cell types including keratinocytes and melanocytes [133,134]. Yang *et al.* generated folliculogenic human epithelial stem cells from human iPSCs that were CD200+ and ITGA6+ and were able to reconstitute all hair follicle lineages and the IFE [135]. Moreover, Tsai *et al.* [136] demonstrated that hair follicle DP cells that endogenously express high levels of Sox2 and c-myc, could be reprogrammed into iPSCs with only Oct4 and Klf4, suggesting that DP cells could be a safer option in iPSC-based therapy. A most recent study suggested that exosomes derived from human induced pluripotent stem cell-derived mesenchymal stem cells (hiPSC-MSCs) facilitate cutaneous wound healing in rats by promotion of collagen synthesis and angiogenesis [137]. These findings highlight the potential use of skin stem cells in iPSC-based therapy, which could be incorporated into tissue-engineered skin scaffolds to generate all cell types, components, and appendages of the skin for the treatment of chronic wounds and other skin disorders. In a recent study, iPSC-derived fibroblasts and keratinocytes from patients with recessive dystrophic epidermolysis bullosa were used to generate 3D skin equivalents and reconstruct human skin structure on the backs of mice [138,139]. More recently, Sebastiano *et al.* [140] generated patient-derived COL7A1-corrected keratinocyte sheets, secreting wild-type type VII collagen, for autologous grafting. These cells formed a stratified epidermis in organotypic cultures and in mice, indicating their potential as a novel therapeutic modality for this devastating skin disease. However, despite experimental evidence supporting the therapeutic benefits of iPSCs, there are some unresolved safety issues that need to be addressed before they can be used in a clinical setting. These include associated cancer risk development through the use of retroviral vectors, inefficient cell re-programming that yields low cell numbers with high processing costs, epigenetic memory retained from parent cells, genetic instability, and potential immunogenicity [141]. To address safety concerns, many new reprogramming techniques are being employed [142] using safer virus-free methods such as chemical compounds, modified RNA, and recombinant proteins [143,144]. Nevertheless, the use of iPSCs surmounts any moral and ethical issues associated with embryonic stem cells. IPSCs therefore hold great promise in the field of wound repair and regenerative medicine as differentiated somatic cells could be isolated from an individual patient and reprogrammed to be differentiated into a desired cell type or a variety of cell types and used in the same patient, circumventing any immunological or rejection issues and, at the same time, promoting patient-tailored treatment.

## 6. Adult Stem Cells and Clinical Applications

A number of the therapies developed for chronic wounds, including negative pressure therapy, hyperbaric oxygen therapy, antimicrobial therapy, bioengineered skin equivalents, maggot debridement therapy, growth factors (reviewed in [9]), have had limited success. Indeed, the topical application of growth factors in an attempt to heal human chronic wounds has been reported with mixed reviews [145], highlighting the complexities of the chronic wound pathology. The drug Regranex, a recombinant human platelet-derived growth factor-BB (rhPDGF-BB), is currently the only growth factor with U.S. Food and Drug Administration (FDA) approval for treatment of DFUs as it has been shown to improve healing in DFUs in randomized clinical trials [146]. Therefore, there is a growing need to explore and develop new treatment strategies to augment chronic wound healing. Further advances in elucidating some of the underlying cellular and molecular mechanisms will be important for the future development of therapies for these difficult-to-treat wounds.

Adult stem cells are now gaining attention in this burgeoning field. To date, many have been isolated such as BMSCs, bone marrow-derived mononuclear stem cells, umbilical cord-derived mesenchymal stem cells (UC-MSCs), adipose-derived stem cells (ASCs), peripheral blood mononuclear cells, placenta-derived stem cells, human fetal aorta-derived progenitor cells, and mesenchymal stem cells (MSCs), with the last of these being well described and most commonly used in preclinical and clinical studies [78,147,148,149,150,151]. Rodriguez-Menocal *et al.* [152] recently demonstrated that among the different bone marrow preparations, cells from whole bone marrow had the greatest positive effects on wound healing both *in vivo* and *in vitro* compared to cultured bone marrow cells or BM-derived MSCs. Some of the ongoing or completed clinical trials registered on www.clinicaltrials.gov are summarized in Table 3. Stem cells have been used successfully to treat both chronic and acute wounds by accelerating wound healing, enhancing re-epithelialization, promoting angiogenesis, exhibiting plasticity, and releasing paracrine signaling molecules [153,154,155]. These cells can be delivered to the wounds either directly (e.g., through spraying, injecting, or systemic administration) or via skin scaffolds. For example, successful delivery of autologous MSCs using a fibrin spray system directly to acute and chronic wounds in mice and humans has been reported [150].

**Table 3:** Clinical trials of stem-cell based therapy for venous ulcers, diabetic foot ulcers, and pressure ulcers on [95].

Conditions	Intervention	Study Phase	ClinicalTrials.gov Identifier
Diabetic foot, venous ulcer, pressure ulcer	Adipose derived stem cells	Phase II	NCT02092870
Diabetic wounds, venous stasis wounds	Lipoaspirate injection	Not available	NCT00815217
Venous ulcer	Autologous bone marrow-derived cell	Phase II	NCT01750749
Critical limb ischemia	Autologous bone marrow stem cell	Phase II	NCT01232673
Diabetic foot, critical limb ischemia	Umbilical cord mesenchymal stem cells	Phase I Phase II	NCT01216865
Diabetic critical limb ischemia	Autologous bone marrow stem cells and tissue repair cells	Phase II	NCT01065337
Diabetic foot, critical limb ischemia, leg ulcers	Granulocyte colony stimulating mobilized autologous peripheral blood mononuclear cell	Phase I Phase II	NCT00922389
Diabetic foot, lower limb ischemia	Autologous mesenchymal stem cells	Phase I	NCT02304588
Type 2 Diabetes Mellitus	Umbilical cord placenta-derived mesenchymal stem cells	Phase I Phase II	NCT01413035
Type 1 and 2 Diabetes Mellitus with foot ulcers	Allogeneic bone marrow-derived mesenchymal stromal cells	Phase I Phase II	NCT01686139
Diabetic foot, venous ulcer, pressure ulcer	Adipose-derived stem cells	Phase II	NCT02092870
Diabetic foot ulcer, critical limb ischemia	Autologous bone marrow mesenchymal stem cells and mononuclear cells	Phase I	NCT00955669
Critical limb ischemia	Autologous bone marrow stem cells	Phase II	NCT01232673
Diabetes, critical limb ischemia	Vascular progenitor cells	Not available	NCT01269580
Diabetic foot, leg ulcer, ischemia	Autologous bone marrow cell concentrate	Phase II Phase III	NCT00434616
Type 2 Diabetes Mellitus	Autologous adipose-derived stem cells	Phase II Phase III	NCT00703612
Diabetic foot ulcer	Autologous endothelial progenitor cells	Not available	NCT02474381
Diabetic foot ulcer	Allogenic adipose-derived mesenchymal stem cells in hydrogel sheet	Phase I	NCT02394886
Lower extremity ischemia, leg ulcer, diabetic foot ulcer	Autologous bone marrow-derived mononuclear cells	Phase I Phase II	NCT01903044
Diabetic foot	Autologous bone marrow mononuclear cells	Phase I Phase II	NCT00872326
Diabetic foot	Intra-arterial infusion of autologous bone marrow cells	Phase I Phase II	NCT00987363
Diabetic foot ulcer, leg ulcers, critical limb ischemia	Granulocyte colony stimulating factor and peripheral blood derived mononuclear cells	Phase I Phase II	NCT00922389

Typically, these scaffolds are seeded with primary fibroblasts and keratinocytes. However, with new data emerging from studies regarding the robustness of adult stem cell types, their incorporation into such scaffolds may prove to be an attractive option for wound therapies. The therapeutic effects of BMSCs seeded on collagen lattices applied to a variety of wound types in patients has been shown to improve wound healing [147]. Badiavas and Falanga reported the successful use of autologous BMSCs embedded in collagen matrices in the treatment of chronic leg ulcers [156]. In another study, exosomes of MSCs were found to be mediators of wound healing [78]. ASCs embedded in AlloDerm and a fibrin–chitosan scaffold have also been shown to augment wound healing by releasing angiogenic factors that contribute to vascular network development [149]. Composite skin equivalents composed of human DP cells derived from scalp skin seeded on collagen type I gel with human neonatal foreskin keratinocytes were grafted onto nude mice and were able to form hair follicles that expressed human nestin and versican, a marker of DP cell inductive ability [157,158]. In another study, reconstituted hair-producing skin using a simplified procedure was produced by recombining a suspension of newborn mouse epidermal and dermal stem cells *in vitro* to form a gel-like matrix or seeding into Integra Artificial Skin before application to full-thickness wounds on the back of mice [151]. The findings from these studies provide evidence for the potential use of stem cells as candidates for cell/scaffold-based novel wound therapeutics.

## 7. Advances in Smart Matrices for Optimal Cell Survival, Preservation, and “Stemness”

Skin scaffolds like dermal matrices promote cell proliferation and regeneration by providing a spatiotemporal environment [159]. They are available in various forms including natural, synthetic, and hybrid matrices and are prepared by various techniques including solid free-form fabrication, electrospinning, phase separation, freeze-drying, and self-assembly (reviewed in [159,160,161,162]). Dermal matrices, which are simple analogues of ECM, can be cellular or acellular, biodegradable or non-biodegradable polymers (Table 2). Natural matrices have associated risks of disease transmission and immunogenicity. Conversely, synthetic matrices can be manufactured in large quantities, are more standardized, thus reducing variability, and carry minimal risk of disease.

Although these skin scaffolds can be used to aid tissue repair and regeneration, they have their limitations and do not replace all the functions of skin nor regenerate skin appendages, even after *in vivo* engraftment. Moreover, cell-seeded matrices have been plagued with low cell proliferation and survival rates and a lack of persistence when used as wound therapeutics [163,164]. In support of this, Griffiths *et al.* [163] demonstrated that the allogeneic cells in Apligraf, an FDA-approved therapeutic product for chronic wounds, did not persist long term *in vivo* and the product itself acted only as a temporary biological dressing, providing growth factors to acute, deep-dermal wounds. Therefore, there is a strong need to seek alternative strategies to optimize cell survival in tissue-engineered scaffolds to improve wound therapeutics. Electrospinning and 3D bioprinting are novel methods used in matrix design to achieve cell-seeding, viability, and scaffold standardization. Studies reported that electrospun scaffolds promote fibroblast viability and maintenance *in vitro* [165], improve cellular organization, and reduce wound contraction compared to freeze-dried scaffolds in a murine full-thickness wound model [166]. Lee *et al.* [167] also demonstrated in a proof-of-concept study the successful use of 3D bioprinting for tissue engineering of human skin in a layer-by-layer assembly process using collagen type 1, fibroblasts, and keratinocytes. The future holds great promise with advances in stem cell biology and techniques for their identification, isolation, and expansion. These cells can potentially be explored in scaffold-based therapeutic strategies to provide novel therapeutic approaches. Pure populations of stem cells in skin scaffolds are likely to promote cell preservation and tissue regeneration. Ghazizadeh and Taichman [168] used a retrovirus-mediated gene transfer technique to genetically mark the epidermal stem cells of adolescent mice, and were able to follow the fate of the marked progeny after 37 epidermal turnovers and five cycles of depilation-induced hair growth. Another study reported a cell sorting method, using a Hoechst and propidium iodide dye combination and specifically defined gating, for the isolation of a pure population of mouse epidermal stem cells that when incorporated in bioengineered skin composed of collagen type I gel seeded with dermal fibroblasts were able to form and maintain a normal epidermis for up to six months in an organotypic culture [82]. Furthermore, Orbay *et al.* [169] reported that the addition of ASCs contributed to the preservation of the engrafted dermal matrix long-term and reduced tissue atrophy. Studies have also explored several approaches to preserving stem cell characteristics and behavior *in vitro* in order to optimize their efficacy when delivered *in vivo*. Rustad *et al.* [170] recently showed that MSCs, when seeded on bioscaffolds composed of a pullulan-collagen lattice, were able to maintain stem cell-related gene expression and enhance wound healing in comparison to MSCs delivered by injection techniques. Hair follicle DP cells lose their aggregative behavior and, hence, their hair follicle inductive ability in culture [171,172,173]. Higgins *et al.* [174] demonstrated that this was a result of major changes in gene expression in DP cells from intact isolated human DP compared to DP cells grown in culture. However, inductive capacity was partially restored when these cells were grown as 3D spheroid cultures and were able to induce hair follicle formation in foreskin epidermis and dermis grafted to nude mice. Other studies have demonstrated the self-assembly of DP cells into inductive spheroidal microtissues when seeded on controlled biomaterial scaffolds like poly (ethylene-*co*-vinyl alcohol) (EVAL) [175] or polyvinyl alcohol (PVA) membranes [176]. Further research into optimizing protocols for stem cell preservation and activity in cell/scaffold-based products will aid in the development of more effective therapies for skin-related conditions.

## 8. Conclusions

In this review, we have discussed epidermal and other skin stem cells and their potential application as cell/scaffold-based stem cell therapies for skin disorders and non-healing wounds. The research community strives to elucidate the roles of adult stem cells, associated molecular pathways, and matrix components in order to restore disturbed skin homeostasis, thus aiding in the future development of more effective therapeutic strategies. Emulating the complex cellular interactions and regulators of stem cell behavior in skin scaffolds, although challenging, remains an important focus. Choosing the right stem cell type that will aid in the complete regeneration of fully functional skin with all components and appendages *in vivo* is extremely important. Furthermore, identifying and isolating pure adult stem cell populations, optimizing protocols for cell-seeding in matrices, and designing scaffold structure composition are all factors that need to be further investigated for the optimization of tissue repair and regeneration. One strategy would be to use a combined approach of stem cells with nanoparticle-containing smart matrices that can mimic the stem cell niche; direct, instruct, and permit stem cell survival and preservation when delivered to the wound to actively promote wound healing; and mimic the intrinsic properties of a native tissue environment. The tailoring of these products to different skin disorders and wound types as a more personalized approach may lead to more effective therapies. Therefore, more clinical trials are required to further explore the long-term effects of using these cells and to ultimately provide safer and more effective therapies for future clinical applications.
